# Efficacy of surgical treatment of perianal infection in patients with hematological malignancy

**DOI:** 10.1097/MD.0000000000038082

**Published:** 2024-05-10

**Authors:** Jie He, Zhijie Ni, Zhongbo Li

**Affiliations:** a Department of Colorectal Surgery, China Aerospace Science & Industry Corporation 731 Hospital, Beijing, China.

**Keywords:** complication, hematologic malignancy, neutropenia, perianal infection, sepsis

## Abstract

The efficacy of surgical intervention for perianal infection in patients with hematologic malignancies is not well established. Therefore, our study aimed to investigate the clinical efficacy and complications of surgical treatment of perianal infection in patients with hematologic malignancies. This retrospective study included patients with hematological malignancies who were diagnosed with perianal infections and treated at the China Aerospace Science & Industry Corporation 731 Hospital between 2018 and 2022. Patient characteristics, hematological data, surgical intervention, and complications, including recurrence and mortality, were analyzed. This study included 156 patients with leukemia aged 2 months to 71 years who were treated surgically for perianal infection, comprising 94 males and 62 females. Perianal infection included 36 cases of abscesses, 91 anal fistulas, and 29 anal fissures accompanied by infection. A total of 36 patients developed severe complications postoperatively, including 4 patients who died, 6 patients with severe incision bleeding, 18 patients with severe pain, 6 patients with sepsis, 12 patients who needed reoperation, 15 patients with hospitalization for more than 2 weeks, and 3 patients with anal stenosis; none of the patients developed anal incontinence. Additionally, risk factors for postoperative complications of perianal infection in patients with hematologic malignancies include leukopenia, agranulocytosis, thrombocytopenia, depth of abscess and not undergone an MRI. Surgical intervention may improve the prognosis of patients with perianal abscess formation, particularly in patients who show no improvement with medical therapy and those who develop perianal sepsis. Granulocytopenia and thrombocytopenia should be improved before surgery, which can significantly reduce postoperative complications. Although these findings are from a case series without a comparator, they may be of value to physicians because to the best of our knowledge, no randomized or prospective studies have been conducted on the management of perianal infections in patients with hematological malignancies.

## 1. Introduction

Patients with acute leukemia belong to a special group as they often present with varying degrees of anemia, bleeding, fever, and infiltration symptoms. Because of their low immunity and the need for repeated and long-term chemotherapy, the incidence of perianal infection is significantly increased (6%–16.8%), especially in patients with neutropenia.^[1,2]^ The mortality rate due to perianal sepsis is high and reportedly between 11% and 57% in patients with neutropenia.^[3]^ However, the clinical signs and symptoms of perianal infections are often camouflaged.^[4]^ Perianal sepsis is a common condition that ranges from acute abscesses to chronic fistula formation. In most cases, the infection source is a nonspecific cryptoglandular infection originating in the inter-sphincteric space.^[5]^ The key to successful treatment is the eradication of the primary tract. However, the clinical evaluation of such patients is challenging, as the clinical signs are often masked due to the lack of a robust immune response to localize the infection and form a clinically recognizable abscess. Additionally, digital rectal examination is often not performed due to the concern of breaching the mucosal barrier and worsening the translocation of bacteria in immunocompromised patients, a concept that is open to debate.^[6]^ Currently, there is no consensus on the clinical evaluation of patients with neutropenia and perianal sepsis. The American Society of Colon and Rectal Surgeons advocates the use of computed tomography (CT) or magnetic resonance imaging (MRI) to better assess the perianal anatomy and underlying abscess.^[7]^ MRI can successfully identify the pathological anatomy in all forms of anorectal disease with an accuracy of up to 90%.^[8]^ Recent evidence suggests that MRI has a role in evaluating the perianal area in patients with hematological malignancy, particularly in patients with suspected complex perianal abscess or fistulous disease.

Controversies exist concerning the management of perianal infections in patients with hematological malignancies, and there is no consensus regarding the treatment protocol.^[9]^ Although some authors consider surgical treatment necessary for patients with neutropenia,^[10]^ others believe that surgical treatment causes an increase in mortality compared to medical treatment.^[11]^ It has been reported that 37% to 58% of these patients are treated surgically, and the neutrophil count is considered an important factor for both treatment and diagnosis. The purpose of this study was to observe the efficacy and safety of surgical treatment for perianal infection in patients with hematologic malignancy.

## 2. Materials and methods

### 2.1. Setting and inclusion criteria

In this study, data from patients with hematologic malignancies who were surgically treated for perianal infections at the China Aerospace Science & Industry Corporation 731 Hospital between January 2018 and December 2022 were retrospectively reviewed. The inclusion criteria were as follows: initial presentation with 1.5 × 10^9^/L < white blood cell count < 10 × 10^9^/L, platelets (PLT) > 30 × 10^9^/L, and hemoglobin (HGB) > 60 g/L; Coagulation dysfunction and electrolyte disorders were effectively corrected; No other systemic infections (respiratory, urinary, and digestive systems) or the infection had been cured; The heart and lung function can tolerate the operation and the patient general condition meets the requirements of the operation without the need for long-term bed rest.

### 2.2. Study outcomes

The primary study outcome was mortality and the secondary outcome was surgical complications (incision bleeding, severe pain, delayed healing or nonunion, recurrence, anal stenosis, etc). The clinical status and management of patients were assessed to determine their association with mortality and morbidity.

### 2.3. Management

With sufficient preoperative preparation,^[12]^ all patients were treated with diverting ostomy and surgical debridement once the diagnosis was confirmed. In patients with acute inflammation, only incision and drainage were performed. Inflammation was controlled preoperatively, and a broad-spectrum antibiotic regimen was administered after the surgery with de-escalation based on the culture results and clinical response.^[13]^ The patients underwent dressing changes every day for 1 week after the operation, and the incisions were kept clean and dry. If necessary, appropriate blood products were infused to maintain the coagulation and nutritional status. All patients underwent the necessary traditional Chinese medicine hip baths daily. After 2 weeks, it was recommended that the patients receive the relevant treatment for the hematologic malignancy as soon as possible. The patients were followed up until one of the outcomes appeared.

### 2.4. Statistical analysis

Data were analyzed using SPSS, version 24.0. Demographic characteristics were analyzed using t-tests for 2 independent samples. Count data are presented as rates and percentages, and were compared using the chi-square and rank-sum tests. Statistical significance was set at *P* < .05.

## 3. Results

The study included 156 patients with leukemia who underwent surgery for perianal infection at the China Aerospace Science & Industry Corporation 731 Hospital. The demographic data of the patients with hematologic malignancies, including disease type and postoperative complications, are shown in Table 1. Their age ranged from 2 months to 71 years. This study included 94 males and 62 females. The cases of perianal infection comprised 36 cases of abscesses, 91 anal fistulas, and 29 anal fissures accompanied by infection. A total of 36 patients (23.1%) developed severe postoperative complications, including 4 patients who died, 6 patients with severe incision bleeding, 18 patients with severe pain, 6 patients with sepsis, 12 patients who needed reoperation, 15 patients with hospitalization for more than 2 weeks, and 3 patients with anal stenosis; there were no patients with anal incontinence. Among the 36 patients who had complications, 25 (69.4%) patients mainly had incision pain, recurrence, and hospitalization time of more than 2 weeks. After active treatment, only 2 patients had a poor prognosis and did not improve.

**Table 1:** Demographic data of patients with hematologic malignancies, including the disease type and postoperative complications.

	N (%)
Age (yr), n (%) 0–20 21–40 41–60 61–80	39 (25.0%) 49 (31.4%) 60 (38.5%) 8 (5.1%)
Sex, n (%) Male Female	94 (60.2%) 62 (39.8%)
Malignancy, n (%) AML Lymphoma ALL Multiple myeloma	85 (54.5%) 12 (7.7%) 56 (35.9%) 3 (1.9%)
Days post-chemotherapy, median (range)	16.4 (7–32)
Type of infection, n (%) Abscess Fistula Fissure	36 (23.1%) 91 (58.3%) 29 (18.6%)
Stem cell transplant recipient, n (%)	17 (10.9%)
Underwent MRI, n (%)	29 (18.5%)
Complication, n (%) Death Severe pain (VAS = 7–9) Severe incisional bleeding Sepsis Recurrence Anal incontinence Anal stenosis Length of stay > 2 wk	36 (23.1%) 4 (2.6%) 18 (11.5%) 6 (3.8%) 6 (3.8%) 12 (7.7%) 0 (0%) 3 (1.9%) 15 (9.6%)

ALL = acute lymphoblastic leukemia, AML = acute myeloid leukemia, VAS = visual analog scale.

In addition, the risk factors for postoperative complications of perianal infection in patients with hematologic malignancies include leukopenia, agranulocytosis, thrombocytopenia, depth of abscess and not undergone an MRI (Table 2).

**Table 2:** Comparison of preoperative factors between the groups with and without postoperative complications.

	Complications (n = 36)	No complications (n = 120)	*P* value
Hematological data, median (range) WBC count (10^9^/L) Hemoglobin level (g/L) Neutrophil count (10^9^/L) Platelet count (10^9^/L) C-reactive protein (mg/L) Albumin level (g/L) PT(S) APTT(S) FIB (g/L) Abscess range > 5 cm, n (%) Depth of abscess > 5 cm, n (%) Complex anal fistula, n (%) Operation time > 30 min, n (%) CT > 4 times, n (%) Underwent MRI, n (%)	2.1 ± 0.9 61.5 ± 10.1 0.8 ± 0.6 47.6 ± 41.3 40.6 ± 34.2 30.8 ± 2.5 12.5 ± 1.6 28.6 ± 2.7 1.7 ± 0.8 10 (27.8%) 6 (16.7%) 9 (25.0%) 21 (58.3%) 16 (44.4%) 10 (27.8%)	5.7 ± 4.3 70 ± 20.3 4.1 ± 3.1 85.2 ± 63.4 20.4 ± 13.4 32.7 ± 3.2 12.5 ± 4.3 29.1 ± 5.3 2.4 ± 1.4 16 (13.3%) 7 (5.8%) 39 (32.5%) 41 (34.1%) 37 (30.8%) 16 (13.3%)	<.05 NS <.05 <.05 NS NS NS NS NS NS <.05 NS NS NS <.05

APTT = activated partial thromboplastin time, CT = chemotherapy, FIB = fibrinogen, MRI = magnetic resonance imaging, PT = prothrombin time, WBC = white blood cells.

## 4. Discussion

Perianal infection occurs in 7.3% to 9% of patients with acute leukemia, and its clinical diagnosis is challenging.^[14]^ There is growing support for the use of MRI for evaluating perianal sepsis in patients with hematological malignancy, as it clearly delineates the perianal anatomy and detects complex perianal diseases, including fistulas, that can inform preoperative planning.^[15]^ In our study, preoperative MRI can reduce the probability of postoperative complications. MRI was mainly used to understand the course and number of sinus tracts, which can avoid intraoperative omissions and lead to the occurrence of complications. The number of granulocytes determines the type of inflammation, course of infection, and likelihood of developing sepsis. In our study, leukopenia, agranulocytosis be considered as independent statistically significant prognostic risk factors for postoperative complications of perianal infection.

As stated earlier, there is no consensus regarding the treatment protocol for the management of perianal infections in patients with hematological malignancies.^[9]^ Some authors have concluded that surgical interventions could yield good results when they are performed for a well-bordered and fluctuant abscess, which is a sign of efficient neutrophil function; if the patient overall condition does not allow, incision and drainage can be performed first, followed by radical surgery later, which is consistent with our observation. Surgery is still necessary for patients with definite perianal infection. We also suggest that granulocyte deficiency should be improved before surgery. Emergency surgery should be avoided in the acute stage of inflammation; otherwise, it may cause spread of inflammation or formation of a false passage. Only incision and drainage should be performed in such cases, and a second surgery should be performed after the formation of an anal fistula. Additionally, 30% to 88% of patients with neutropenia, but without a fluctuating abscess with clear borders were successfully treated with medical therapy alone.

Furthermore, we have the following suggestions for improving the postoperative treatment: Active anti-infection measures: once a perianal infection is detected, highly effective broad-spectrum antibiotics should be administered as early as possible, following the principle of descending the ladder of drug use. Hydrocarbon enzyme alkene antibiotics can be applied empirically, and the antibiotics should be adjusted according to the bacteriological results of anal swabs, pus cultures, blood cultures, etc. Clinical studies have shown that an increasing number of infections are caused by gram-positive cocci, which may be closely related to the extensive preventive use of antibiotics and repeated puncture.^[11]^ Blood transfusion: According to the patient hematological malignancy, suspended red blood cells, platelets, plasma, fibrinogen, albumin, and other blood products should be administered to correct anemia, thrombocytopenia, coagulation dysfunction, and hypoproteinemia. Wound treatment: The surgical incision should be kept clean and dry, the wound should be thoroughly drained, and no dead space should remain. Sitting baths with traditional Chinese medicine and local red light irradiation can effectively relieve the local symptoms. If a patient has severe pain, it is necessary to use analgesics to relieve pain. Recovery of gastrointestinal function after surgery: The patient is often malnourished due to the underlying disease, and perianal infections and abscess often have a large basal metabolism; therefore, the patients should have high-calorie food, supplemented with adequate proteins and vitamins. Patients should be encouraged to drink more water and eat light and easily digestible food as much as possible, while avoiding spicy, irritating, and gas-producing foods. Eating fresh fruits and vegetables is also recommended to avoid aggravation of inflammation, in addition to promoting smooth bowel movements. On the first day after surgery, depending on the patient previous bowel movements, appropriate treatment should be administered to moisten the stools and relieve constipation. Intestinal probiotic drugs can be added to improve the intestinal environment. Psychological nursing: Patients often experience different degrees of anxiety, pessimism, fear, and other negative emotions during the long-term treatment of hematological malignancy. While treating these patients, health professionals should communicate with them about their condition, give them support and encouragement, enhance their confidence in overcoming the disease, and treat them both physically and mentally, thus improving their compliance. Early hematological malignancy-related treatment: In our study, approximately 2 weeks after the operation, the patients’ infection was controlled, and hematological malignancy-related treatment was performed without complete healing of the incision.

This study has some limitations. First, this was a case series without a comparator. Second, no standardized approach was used to treat perianal infections in patients with hematologic malignancies. Lastly, only patients with hematological malignancies who underwent surgery for perianal infections were analyzed.

The diagnosis and management of perianal infections are difficult in patients with hematological malignancies. High clinical suspicion may effectively reduce mortality by allowing early intervention and close monitoring, particularly in patients with fever accompanied by anal pain. Collaborative communication between subspecialists may aid in the diagnosis. Surgery may improve the prognosis of patients with perianal inflammation extending beyond the perianal region, abscess formation, or no improvement with medical therapy.

## Author contributions

**Conceptualization:** Jie He.

**Data curation:** Zhijie Ni.

**Formal analysis:** Jie He.

**Methodology:** Jie He.

**Validation:** Jie He.

**Writing – original draft:** Jie He.

**Writing – review & editing:** Zhongbo Li.
